# Association Between Maternal Serum 25-Hydroxyvitamin D Concentrations and Gestational Diabetes Mellitus in Women Undergoing Diagnostic 100-g Oral Glucose Tolerance Testing

**DOI:** 10.3390/jcm15155977

**Published:** 2026-07-31

**Authors:** Hüseyin Karakaya, Gökhan Doğukan Akarsu, Taylan Onat, Rukiye Höbek Akarsu

**Affiliations:** 1 Department of Gynecology and Obstetrics, Faculty of Medicine, Yozgat Bozok University, Yozgat 66100, Türkiye; drhusyn@gmail.com; 2 Department of Pharmacy Services, Vocational School of Health Services, Yozgat Bozok University, Yozgat 66100, Türkiye; 3 Department of Gynecology and Obstetrics, Sincan Training and Research Hospital, Ankara 06949, Türkiye; onat.taylan@gmail.com; 4 Department of Nursing, Faculty of Health Sciences, Yozgat Bozok University, Yozgat 66100, Türkiye; rukiye-hobek@hotmail.com

**Keywords:** SDG 3, gestational diabetes mellitus, vitamin D, pregnancy, insulin resistance, PLR

## Abstract

**Background:** Gestational diabetes mellitus (GDM) is a common metabolic complication of pregnancy. Vitamin D has been implicated in glucose metabolism; however, its association with GDM remains controversial. **Objective:** The objective was to evaluate the association between maternal serum 25-hydroxyvitamin D [25(OH)D] concentrations and GDM in pregnant women referred for diagnostic 100 g oral glucose tolerance testing following a positive 50 g glucose challenge test. **Methods:** This retrospective study included 92 pregnant women (44 with GDM and 48 without GDM) who underwent diagnostic 100 g oral glucose tolerance testing between January 2016 and June 2025 at a tertiary-care hospital. Demographic characteristics and biochemical parameters, including serum 25(OH)D concentrations, were obtained from medical records. Logistic regression analysis was performed to identify factors independently associated with GDM. **Results:** Women with GDM were significantly older than those without GDM (37.55 ± 6.06 vs. 29.21 ± 4.29 years, *p* < 0.001) and had lower serum vitamin D concentrations (14.08 ± 9.88 vs. 19.06 ± 10.30 ng/mL, *p* = 0.003). In multivariable logistic regression analysis, advanced maternal age (OR 1.404, 95% CI 1.217–1.620; *p* < 0.001) and lower serum vitamin D concentrations (OR 0.933, 95% CI 0.880–0.989; *p* = 0.020) were independently associated with GDM. **Conclusion:** Lower maternal serum 25(OH)D concentrations were independently associated with GDM in women referred for diagnostic 100 g oral glucose tolerance testing. These findings should be interpreted as observational and hypothesis-generating. Larger prospective studies are needed to confirm these results.

## 1. Background

Gestational diabetes mellitus (GDM) is one of the most common metabolic complications of pregnancy and is associated with adverse maternal and fetal outcomes. Its prevalence has been increasing worldwide, largely due to advanced maternal age and the rising prevalence of obesity [1]. Diabetes mellitus is a leading cause of morbidity and mortality among non-communicable diseases and represents a growing global public health burden [2].

Routine screening for gestational diabetes mellitus during pregnancy enables early diagnosis and appropriate management, which may reduce the risk of adverse maternal and fetal outcomes such as macrosomia, shoulder dystocia, polyhydramnios, increased cesarean delivery rates, and fetal loss [3,4]. The prevalence of GDM varies across populations depending on ethnicity and geographic region, affecting up to 20% of pregnancies [5]. Established risk factors include advanced maternal age, family history of diabetes, pre-pregnancy body mass index, and lifestyle-related factors [6]. The impact of GDM extends beyond pregnancy, with both maternal and offspring consequences persisting into the postpartum period. Women with a history of GDM have an increased risk of developing type 2 diabetes and cardiovascular disease, while offspring are at higher risk of obesity and metabolic disorders later in life [7]. Vitamin D is a fat-soluble hormone with autocrine, endocrine, and paracrine functions. Beyond its well-established role in bone metabolism, it has been increasingly recognized for its involvement in various metabolic and cardiovascular conditions, including diabetes, metabolic syndrome, and hypertension [8,9,10,11,12,13].

Many studies have demonstrated an association between vitamin D deficiency and both type 1 and type 2 diabetes. However, evidence regarding its role in gestational diabetes mellitus remains limited and inconsistent [14,15,16,17]. Several mechanisms play a role in GDM development. These include insulin resistance, β-cell dysfunction, adipose tissue dysfunction, gluconeogenesis, and oxidative stress. Because of this complexity, the exact cause of GDM remains unclear [18,19].

Vitamin D deficiency has been proposed as a potential factor associated with GDM and may help identify women at increased risk before pregnancy. However, the available evidence remains inconsistent. This study aimed to evaluate the association between maternal serum 25-hydroxyvitamin D [25(OH)D] concentrations and gestational diabetes mellitus among pregnant women referred for diagnostic 100 g oral glucose tolerance testing following a positive 50 g glucose challenge test.

## 2. Material and Method

### 2.1. Study Groups

This retrospective study was conducted at the Obstetrics and Gynecology outpatient clinic of a tertiary-care hospital. Medical records of pregnant women who underwent a 100 g oral glucose tolerance test (OGTT) between January 2016 and June 2025 were reviewed. Participants were divided into two groups based on GDM status: those diagnosed with GDM and those without GDM (control group). The control group consisted of women referred for diagnostic 100 g oral glucose tolerance testing following a positive 50 g glucose challenge test who did not meet the Carpenter–Coustan diagnostic criteria for GDM. Therefore, the comparison group does not represent an unselected obstetric population but rather women with abnormal screening results who were subsequently found not to have GDM. Inclusion criteria were age between 18 and 45 years and absence of known chronic diseases. Demographic characteristics and routine biochemical parameters, including 25(OH)D concentrations, were extracted from medical records and entered into a statistical database.

Routine screening for gestational diabetes mellitus was performed between 24 and 28 weeks of gestation using a 50 g oral glucose challenge test. Pregnant women with a 1 h plasma glucose level of ≥140 mg/dL underwent a diagnostic 100 g oral glucose tolerance test (OGTT). Gestational diabetes mellitus was diagnosed according to the Carpenter–Coustan criteria, whereby the presence of at least two abnormal plasma glucose values exceeding the threshold levels of 95 mg/dL (fasting), 180 mg/dL (1 h), 155 mg/dL (2 h), and 140 mg/dL (3 h) was considered diagnostic. Serum 25(OH)D concentrations were measured using a fully automated hormone immunoassay analyzer as part of routine clinical care. Although the date of serum 25(OH)D measurement was available in the medical records, the exact gestational week at the time of blood sampling and information regarding vitamin D supplementation were not consistently documented because of the retrospective study design.

### 2.2. Statistical Analysis

Statistical analyses were performed using IBM SPSS Statistics version 25 (IBM Corp., Armonk, NY, USA). Continuous variables were expressed as mean ± standard deviation or median (minimum–maximum), while categorical variables were presented as numbers and percentages. The normality of data distribution was assessed using the Shapiro–Wilk test. For normally distributed variables, comparisons between two groups were performed using the independent samples *t*-test, while non-normally distributed variables were analyzed using the Mann–Whitney U test. Categorical variables were compared using the chi-square test. Logistic regression analysis was performed to identify independent factors associated with GDM. Variables with a *p*-value < 0.20 in the univariate analyses were considered for inclusion in the multivariable logistic regression model. Maternal age, platelet count, vitamin D level, monocyte count, and lymphocyte count were entered into the final model. Parity, number of living children, and delivery type were excluded because of sparse cell counts and concerns regarding model stability. Odds ratios (ORs) with 95% confidence intervals (CIs) were calculated, and a *p*-value < 0.05 was considered statistically significant.

## 3. Results

A total of 92 pregnant women were included in the study, comprising 44 women with GDM and 48 without GDM. The mean age was significantly higher in the GDM group compared to the non-GDM group (37.55 ± 6.06 vs. 29.21 ± 4.29 years, *p* < 0.001). Gravida status did not differ significantly between groups (*p* > 0.05). Nulliparity was significantly more common in the non-GDM group (*p* < 0.001). A history of abortion was more frequent among women with GDM (*p* < 0.05) (Table 1).

No significant differences were observed between the groups in leukocyte, neutrophil, hemoglobin, lymphocyte, MPV, and NLR levels (*p* > 0.05). However, platelet count, vitamin D levels, monocyte levels, and PLR were significantly lower in the GDM group compared to the non-GDM group (*p* < 0.05) (Table 2).

Multivariable logistic regression analysis demonstrated that maternal age and serum vitamin D level remained independently associated with GDM after adjustment for the other variables included in the model. Platelet count, monocyte count, and lymphocyte count were not independently associated with GDM (Table 3).

## 4. Discussion

In this study, we evaluated the association between maternal serum 25-hydroxyvitamin D [25(OH)D] concentrations and gestational diabetes mellitus in pregnant women referred for diagnostic 100 g oral glucose tolerance testing following a positive 50 g glucose challenge test. Our findings showed that lower vitamin D levels were significantly associated with the presence of GDM. In addition, advanced maternal age was independently associated with GDM, while lower PLR values were observed in women with GDM in the univariate analysis. Maternal age was significantly higher in the GDM group, which is consistent with previous reports identifying advanced maternal age as a major risk factor for GDM [20]. Age-related changes such as reduced insulin sensitivity, increased visceral adiposity, and chronic low-grade inflammation contribute to the development of insulin resistance during pregnancy. In addition, the decline in pancreatic β-cell function with age may limit the ability to compensate for the increased metabolic demands of pregnancy. These mechanisms likely explain the higher prevalence of GDM observed in older pregnant women.

Multiparity has also been linked to increased metabolic burden and a higher risk of metabolic disorders [21,22]. Repeated pregnancies may lead to cumulative weight retention, increased central adiposity, and persistent alterations in glucose metabolism. These changes can predispose women to insulin resistance and impaired glucose tolerance over time. In our study, GDM was more frequently observed in multiparous women. However, because parity was not included in the final multivariable model owing to model instability, this finding should be interpreted with caution.

In this study, PLR levels were significantly lower in the GDM group in univariate analysis. However, PLR was not retained in the final multivariable model because of model instability. Therefore, whether PLR serves as an independent predictor of GDM warrants further investigation in larger prospective studies. Platelets are not only involved in hemostasis but also play a role in inflammatory processes. GDM is characterized by increased oxidative stress and low-grade inflammation, which may influence platelet activation and related indices [23,24]. Previous studies evaluating inflammatory markers in GDM have reported inconsistent results. For example, a meta-analysis by Hessami et al. demonstrated that NLR was significantly elevated in GDM patients, whereas PLR did not show a consistent association [25]. In contrast, our findings indicate that lower PLR values may be associated with increased GDM risk. This discrepancy may be explained by differences in study populations, gestational age at sampling, ethnic background, or underlying metabolic characteristics. Further studies are needed to clarify the role of PLR as a potential biomarker in GDM.

The role of vitamin D in glucose metabolism has been increasingly investigated in recent years. Vitamin D is known to influence pancreatic β-cell function, insulin secretion, and peripheral insulin sensitivity [26]. In addition, it may modulate inflammatory pathways and oxidative stress, both of which are implicated in the pathogenesis of GDM. Observational studies have reported that low serum 25(OH)D levels are associated with an increased risk of GDM [27,28]. These findings are in agreement with previous observational evidence suggesting that lower maternal serum 25(OH)D concentrations are associated with an increased risk of GDM. A recent meta-analysis by Milajerdi et al. also reported a modest association between vitamin D deficiency and GDM. However, Mendelian randomization analyses have not supported a robust causal relationship and have suggested that this association may be partly explained by confounding factors, particularly adiposity. Furthermore, recent clinical guidelines do not recommend routine vitamin D screening during pregnancy in the absence of established clinical indications. Therefore, our findings should be interpreted as observational and hypothesis-generating rather than evidence supporting routine vitamin D screening or preventive intervention [29,30,31].

As this study is observational in nature, it is not possible to establish a causal relationship between vitamin D deficiency and GDM. The observed association should be interpreted within the limitations of the study design. Randomized controlled trials evaluating the effect of vitamin D supplementation on GDM risk have produced conflicting results [32,33,34,35,36,37]. While some studies suggest potential benefits, others have not demonstrated a significant reduction in GDM incidence. These inconsistencies may be due to differences in study design, baseline vitamin D status, supplementation dose, and timing of intervention. Therefore, although vitamin D deficiency appears to be associated with GDM, it cannot yet be considered a definitive causal factor. Importantly, the association between lower maternal vitamin D levels and GDM remained statistically significant after adjustment for maternal age and the other variables included in the multivariable model, suggesting that this relationship was not solely explained by differences in maternal age. However, this finding should be interpreted with caution, as several established risk factors for GDM, including pre-pregnancy BMI, gestational weight gain, family history of diabetes, previous GDM, smoking status, ethnicity, and socioeconomic status, were unavailable in this retrospective dataset and therefore could not be accounted for in the multivariable analysis. In particular, adiposity is closely linked to both vitamin D status and insulin resistance, and recent evidence suggests that changes in body composition may contribute substantially to metabolic risk beyond BMI alone [31]. Therefore, residual confounding related to adiposity cannot be excluded.

Taken together, our findings further support the multifactorial nature of GDM and reinforce the observed association between lower maternal vitamin D levels and GDM. Given the observational nature of this study, these findings should be interpreted as evidence of association rather than causation. Because the present study included only women referred for diagnostic 100 g oral glucose tolerance testing following a positive 50 g glucose challenge test, the findings should be interpreted within this selected study population and should not be generalized to the overall pregnant population. Moreover, the observed difference in PLR levels between groups in univariate analysis suggests that inflammatory markers may warrant further investigation as potential biomarkers associated with GDM. Nevertheless, this study has several limitations. The retrospective design limits the ability to establish causality, and the relatively small sample size may affect the generalizability of the findings. In addition, potential confounding factors such as dietary habits, sunlight exposure, and vitamin D supplementation were not evaluated. Future large-scale prospective cohort studies and randomized controlled trials are warranted to better understand the relationship between vitamin D and GDM and to determine whether vitamin D assessment or supplementation has a role in clinical practice. Future studies with larger cohorts should further investigate the relationship between maternal serum 25(OH)D concentrations and clinical characteristics using correlation and dose–response analyses.

## 5. Conclusions

In conclusion, this study demonstrated an independent association between lower maternal serum 25-hydroxyvitamin D [25(OH)D] concentrations and gestational diabetes mellitus in women referred for diagnostic 100 g oral glucose tolerance testing following a positive 50 g glucose challenge test. These findings should be interpreted as observational and hypothesis-generating and should not be generalized to the overall obstetric population. Although PLR levels were lower in women with GDM, this association was not confirmed in the multivariable analysis and therefore requires validation in larger prospective studies. Further well-designed prospective studies are warranted to confirm these findings and clarify the potential role of vitamin D in GDM.

## 6. Limitations

This study has several limitations. First, its retrospective design limits the ability to establish a causal relationship between vitamin D deficiency and GDM. Second, the relatively small sample size may affect the generalizability of the findings, although an a priori power analysis confirmed adequate statistical power for the primary comparisons. Third, the number of variables included in the multivariable logistic regression model was relatively high in relation to the sample size, increasing the risk of model overfitting. Therefore, the regression findings should be interpreted as exploratory and require validation in larger independent cohorts. Fourth, although PLR differed significantly between groups in the univariate analysis, it was not retained in the final multivariable model because of model instability; therefore, its role as an independent predictor of GDM remains uncertain. Fifth, information regarding pre-pregnancy BMI, gestational weight gain, family history of diabetes, previous GDM, smoking status, ethnicity, socioeconomic status, vitamin D supplementation, dietary habits, sunlight exposure, and the exact gestational week at the time of serum 25(OH)D measurement was not consistently available because of the retrospective study design. Although the date of serum 25(OH)D measurement was available, the lack of gestational age information precluded precise assessment of the temporal relationship between vitamin D status and GDM and may have introduced residual confounding. Finally, as this was a single-center study conducted in a small provincial setting, and because the comparison group consisted of women referred for diagnostic 100 g oral glucose tolerance testing following a positive 50 g glucose challenge test, the findings may not be generalizable to an unselected obstetric population. Additionally, MPV measurements were available for only a limited number of participants in the non-GDM group, which may have contributed to the high standard deviation observed for this parameter.

## Figures and Tables

**Table 1:** Comparison of certain characteristics between the study groups.

Characteristics		GDM Positive (*n* = 44) n%		GDM Negative (*n* = 48) n%		Test	*p*
Age (years)	Mean ± SD	37.55 ± 6.06		29.21 ± 4.29		***t*^2^** ***= 7.550***	***<0.001***
Gravida	Primigravida	12	35.3	22	64.7	*X***^2^** *= 2.644*	*0.105*
	Multigravida	32	55.2	26	44.8		
Parity	Nullipar	2	4.9	39	95.1	***X*^2^** ***= 56.249***	***<0.001***
	Primipar	20	74.1	7	25.9		
	Multipar	22	91.7	2	8.3		
Abortion	No	30	41.7	42	58.3	***X*^2^** ***= 3.964***	***0.046***
	Yes	14	70	6	30		
Living children	No	2	7.1	26	92.9	***X*^2^** ***= 24.406***	***<0.001***
	Yes	42	65.6	22	34.4		
Previous mode of delivery	No previous birth	0	0	27	100	***X*^2^** ***= 36.500***	***<0.001***
	Vaginal delivery	10	55.6	8	44.4		
	C-section	34	72.3	13	27.7		

t: Independent *t*-test; X^2^: Chi-square test.

**Table 2:** Comparison of biochemical parameters between the study groups.

Characteristics	GDM Positive (*n* = 44) Mean ± SD	GDM Negative (*n* = 48) Mean ± SD	Test	*p*
Leukocyte (×10^3^/μL)	9.40 ± 3.23	9.30 ± 3.69	*t*^1^ = 1045.50	0.935
Neutrophil (×10^3^/μL)	6.65 ± 3.00	7.92 ± 10.45	*t*^1^ *= 0.646*	*0.644*
Hemoglobin (g/dL)	12.60 ± 1.40	12.47 ± 1.23	*t*^2^ *= 1036.00*	*0.876*
Platelet (×10^3^/μL)	229.27 ± 62.54	259.04 ± 60.58	***t*^2^** ***= −2.322***	***0.022***
Vitamin D (ng/mL)	14.08 ± 9.88	19.06 ± 10.30	***t*^1^** ***= 655.00***	***0.003***
Lymphocyte (×10^3^/μL)	2.29 ± 0.72	2.71 ± 3.36	*t*^1^ *= 885.00*	*0.181*
Monocyte (×10^3^/μL)	0.65 ± 0.74	1.05 ± 1.90	***t*^1^** ***= 777.00***	***0.041***
MPV (fL)	10.54 ± 1.05	24.60 ± 29.87 *	*t*^1^ *= 1008.00*	*0.707*
NLR	3.06 ± 1.43	3.27 ± 1.60	*t*^1^ *= 915.00*	*0.27*
PLR	106.49 ± 35.27	130.97 ± 48.20	***t*^2^** ***= −2.759***	***0.007***

1: Mann–Whitney U test, 2: Independent *t*-test, Mean ± SD: mean ± standard deviation. * MPV measurements were available for only a limited number of women in the non-GDM group; therefore, this parameter should be interpreted with caution.

**Table 3:** Multivariable logistic regression analysis of variables associated with GDM.

Variables	B	Wald	*p*	Exp (B)	% 95.0 CI for EXP(B)	
					Lower	Upper
Age (years)	0.339	21.605	***<0.001***	1.404	1.217	1.62
Platelet	−0.007	1.978	0.16	0.993	0.982	1.003
Vitamin D	−0.069	5.393	***0.02***	0.933	0.88	0.989
Monocyte	0.097	0.165	0.685	1.102	0.691	1.757
Lymphocyte	−0.042	0.092	0.762	0.959	0.732	1.257
Constant	−8.068	9.466	***0.002***	0		

## Data Availability

The datasets generated and/or analyzed during the current study are not publicly available but are available from the corresponding author upon reasonable request and with permission from the relevant institutional authority.
